# Association Between Sarcopenia and Depressive Symptoms in Chinese Older Adults: Evidence From the China Health and Retirement Longitudinal Study

**DOI:** 10.3389/fmed.2021.755705

**Published:** 2021-11-17

**Authors:** Ke Gao, Wen-Zhuo Ma, Scarlett Huck, Bo-Lin Li, Li Zhang, Jiao Zhu, Tian Li, Dan Zhou

**Affiliations:** ^1^Department of Cardiology, The First Affiliated Hospital of Xi'an Jiaotong University, Xi'an, China; ^2^Department of Biomedical and Pharmaceutical Sciences, College of Pharmacy, University of Rhode Island, South Kingstown, RI, United States; ^3^Department of Pharmacology, School of Basic Medicine Sciences, Xi'an Jiaotong University Health Science Center, Xi'an, China; ^4^Department of Gastroenterology, The First Affiliated Hospital of Xi'an Jiaotong University, Xi'an, China; ^5^School of Basic Medicine, Fourth Military Medical University, Xi'an, China

**Keywords:** sarcopenia, possible sarcopenia, depressive symptoms, low muscle mass, older adults

## Abstract

**Background:** Little is known about whether sarcopenia predicts incident depressive symptoms in older adults. Using the nationally representative data from the China Health and Retirement Longitudinal Study (CHARLS), we conducted cross-sectional and longitudinal analyses to estimate the association between sarcopenia and depressive symptoms among older adults.

**Methods:** The sample comprised 7,706 participants aged at least 60 years (50.6% women; mean age 68.0 ± 6.5) from the CHARLS 2015. Based on the Asian Working Group for Sarcopenia 2019 (AWGS 2019) criteria, sarcopenia status was classified into three types: no-sarcopenia, possible sarcopenia, and sarcopenia. Depressive symptoms were assessed using the validated 10-items of the Center for Epidemiologic Studies Depression Scale. A cross-sectional analysis was used to examine the relationship between sarcopenia status and depressive symptoms. A total of 4,652 participants without depressive symptoms were recruited from the same cohort in 2015 and were followed up in 2018. Cox proportional hazards regression models were conducted to examine the effect of sarcopenia status on subsequent depressive symptoms with the report of hazard ratio (HR).

**Results:** The prevalence of depressive symptoms in total populations, no-sarcopenia, possible sarcopenia, and sarcopenia individuals were 27.1% (2085/7706), 21.5% (927/4310), 33.6% (882/2627), and 35.9% (276/769), respectively. Both possible sarcopenia (OR: 1.75, 95% CI: 1.46–2.10) and sarcopenia (OR: 1.64, 95% CI: 1.23–2.19) were positively associated with higher odds of depressive symptoms (all *p* < 0.01). During the 3.7 years of follow-up, 956 cases (20.6%) with incident depressive symptoms were identified. In the longitudinal analysis, individuals with the diagnosed possible sarcopenia (HR: 1.27, 95% CI: 1.01–1.58) and sarcopenia participants (HR: 1.49, 95% CI: 1.06–2.09) were more likely to have new onset depressive symptoms than no-sarcopenia peers.

**Conclusions:** Both possible sarcopenia and sarcopenia, assessed using the AWGS 2019 criteria, were independent predictors for the occurrence of depressive symptoms among Chinese older adults. Our findings provided new evidence supporting the longitudinal connection between sarcopenia and mental health problems, it also provides further justification for timely identification and management of both possible sarcopenia and sarcopenia as part of comprehensive strategies to fight against depressive symptoms.

## Introduction

Sarcopenia, a serious geriatric syndrome with a progressive skeletal muscle disorder, is a public health concern faced by aging societies (1, 2). In 2010, the European Working Group on Sarcopenia in Older People (EWGSOP) first recommended a diagnostic algorithm for sarcopenia (3). In 2014, the Asian Working Group for Sarcopenia (AWGS) consensus defined sarcopenia as “age-related loss of muscle mass, plus low muscle strength, and/or low physical performance” and specified cutoffs for each component (4). According to the AWGS 2014 criteria, the prevalence of sarcopenia among the elderly population ranges from 6.8 to 25.7% in Asian countries (5). Compelling evidence shows that sarcopenia is strongly associated with adverse outcomes, such as falls, fracture, frailty, morbidity, mortality, and increasing healthcare utilization (1, 6–9). To date, the clinical and research interest in sarcopenia has grown rapidly; however, the fields of sarcopenia and routine clinical practice are still largely separated. Most clinicians remain unaware of the condition and the diagnostic methods needed to identify it (1, 10). Therefore, developing and implementing comprehensive strategies to identify sarcopenia and its effects on mental and physical health is crucial.

Depression, a major mental health disorder, is closely linked to an increased likelihood of various health problems among older adults, such as disability, chronic illness (e.g., heart disease, diabetes mellitus, and hypertension), and all-cause mortality (11–14). Several previous studies have shown that sarcopenia was positively correlated with depression and depressive symptoms (15–18). A national survey in China reported that the prevalence of depressive symptoms among Chinese adults aged 45 years and older was 37% in 2015; only <5% of those with depressive symptoms were aware of their conditions and <2% sought care over time (19). Considering the various adverse outcomes associated with depressive symptoms in older people, it is important to understand its associations with sarcopenia. Recently, a growing body of literature has evaluated the relationship between sarcopenia and depressive symptoms (18, 20–22), but almost all studies were cross-sectional, with small sample sizes or using regional data. To date, there have not been large population-based perspective surveys on the causal relationship between sarcopenia and incident depressive symptoms in older adults. To promote healthy aging, the AWGS 2019 introduces “possible sarcopenia,” defined by either low muscle strength or low physical performance only, to enable earlier lifestyle interventions (5). To our knowledge, no studies have evaluated the relationship between possible sarcopenia and depressive symptoms in Asia. Regarding sarcopenia components, in addition to muscle strength and physical performance, the impact of low muscle mass alone on mental health remains to be further studied.

At present, the contribution of individual sarcopenia status to depressive symptoms is still unknown, which gives us the impetus to investigate associations between possible sarcopenia, sarcopenia, and depressive symptoms among older adults in China. Using the nationally representative data from the China Health and Retirement Longitudinal Study (CHARLS) 2015 and 2018, we conducted cross-sectional and longitudinal analyses to estimate the association between sarcopenia status and depressive symptoms among Chinese community-dwelling older adults.

## Methods

### Study Population

The CHARLS, established in 2011, is an ongoing nationally representative longitudinal survey in China. In short, CHARLS collects high-quality data through one-to-one interviews with a structured questionnaire, from a nationally representative sample of the Chinese middle-aged and older population, selected using multistage stratified probability-proportionate-to-size sampling. All participants underwent an assessment using a standardized questionnaire to collect data on sociodemographic and lifestyle factors and health-related information. In CHARLS 2011, a total of 17,708 participants in 10,257 households were recruited from 150 counties or districts and 450 villages within 28 provinces in China. The data included individual weighting variables to ensure that the survey sample was nationally representative. All participants were followed up with every 2 years after the baseline survey. Further details about the study design of the CHARLS have been previously reported (23).

**Figure 1:**
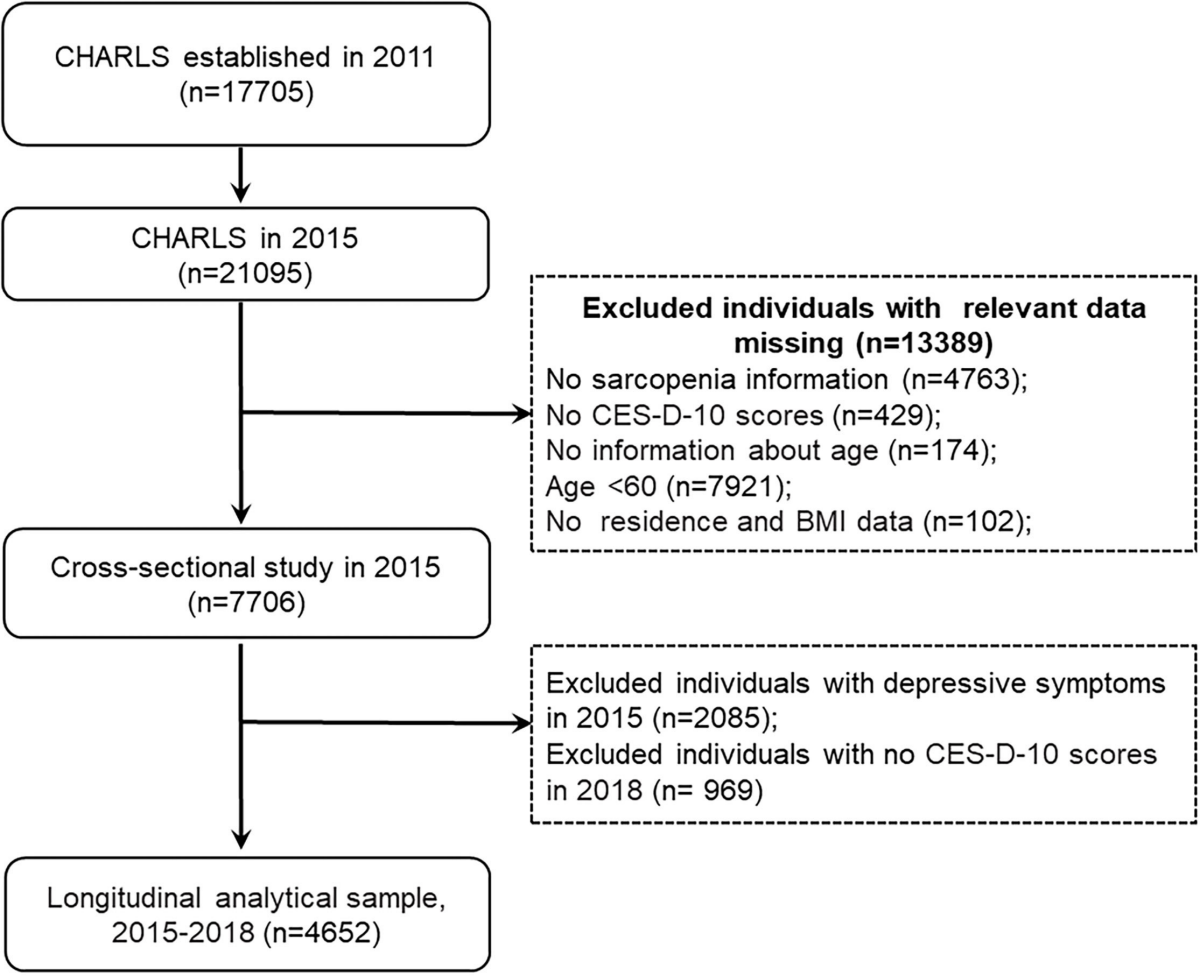
Flow diagram for participants in a cross-sectional and longitudinal study. CES-D-10, the 10-item Center for Epidemiologic Studies Depression Scale; BMI, body mass index.

In our study, we used data from the CHARLS 2015 and 2018. The inclusion criteria for the present study were (1) individuals aged at least 60 years old in CHARLS 2015; (2) having data regarding sarcopenia status. Exclusion criteria were (1) missing data of sarcopenia status; (2) no CES-D-10 scores; (3) no information about age; (4) persons aged <60 years old; and (5) no residence and body mass index data. This study was divided into two sections. (1) In the cross-sectional analysis, we used data from the large cohort that was followed up in 2015. A total of 21,095 participants were interviewed in CHARLS 2015, 13,389 individuals were excluded because of missing data of sarcopenia (*n* = 4,763), no CES-D-10 scores (*n* = 429), no information about age (*n* = 174), aged <60 years (*n* = 7,921), and no residence and body mass index (*n* = 102), leaving 7,706 participants for cross-sectional analysis. (2) In the longitudinal analysis, we further excluded 2,085 subjects with depressive symptoms in CHARLS 2015 and 969 participants without information of CES-D-10 scores in CHARLS 2018. Our final analytic sample included 4,652 participants, who had no depressive symptoms in CHARLS 2015 and followed up in 2018. The detailed selection process was shown in Figure 1.

The Biomedical Ethics Review Committee of Peking University approved CHARLS (approval number: IRB00001052-11015), and all participants were required to provide written informed consent.

### Assessment of Sarcopenia Status

Sarcopenia status was assessed according to the AWGS 2019 algorithm, which is composed of three components: muscle strength, appendicular skeletal muscle mass (ASM), and physical performance (5). Handgrip strength (kg) was measured in the dominant hand and non-dominant hand, with the participant squeezing a YuejianTM WL-1,000 dynamometer (Nantong Yuejian Physical Measurement Instrument Co., Ltd., Nantong, China) as hard as possible (23). Every person was measured two times for both hands by holding the dynamometer at a right angle (90°). The cutoff points for low grip strength for men and women were <28 and <18 kg, respectively. The muscle mass was estimated by ASM using a previously validated anthropometric equation in Chinese residents (24, 25). Several studies have shown that the agreement of the ASM equation model and dual X-ray absorptiometry (DXA) was strong (24, 25). The cutoff for defining low muscle mass was based on the sex-specific lowest 20% of the height-adjusted muscle mass (ASM/Ht^2^) among the study population (25–27). Finally, the ASM/Ht^2^ values of <5.69 kg/m^2^ in women and <6.88 kg/m^2^ in men were considered as low muscle mass. In terms of physical performance, the gait speed and the chair stand test were evaluated using the method described by Wu et al. (27). Using the cutoff points from the AWGS 2019 consensus, low physical performance was defined as a five-time chair stand test ≥12 s, or gait speed on a 6-m walk <1.0 m/s, or Short Physical Performance Battery (SPPB) score <9 (5). Further details about the definitions for sarcopenia components in the CHARLS have been previously published (27).

Sarcopenia is defined when low muscle mass plus low muscle strength or low physical performance are detected. Possible sarcopenia is diagnosed by low muscle strength with or without reduced physical performance. Individuals with all three components, including low muscle mass, low muscle strength, and low physical performance, are considered as having severe sarcopenia (5). In our study population, only 221 (2.9%) participants had severe sarcopenia. Therefore, we merged participants with severe sarcopenia into the sarcopenia group and divided all participants into three groups: no-sarcopenia, possible sarcopenia, and sarcopenia.

### Assessment of Depressive Symptoms

In the CHARLS 2015 and 2018, depressive symptoms were measured using the 10-item short form of the Center for Epidemiologic Studies Depression Scale (CES-D-10) (28), which is a widely used self-report measure on depressive symptoms in population-based studies. The CES-D-10 has satisfactory validity and excellent psychometric properties among Chinese older individuals (29). The CES-D short form consists of 10 items: (1) bothered by little things, (2) had trouble concentrating, (3) felt depressed, (4) everything was an effort, (5) felt hopeful, (6) felt fearful, (7) sleep was restless, (8) felt happy, (9) felt lonely, and (10) could not get going. Each depressive symptom item in the past week was measured from 0 (rarely or none of the time [ <1 day]) to three points (most or all of the time [5–7 days]). Individuals who had scores of at least 12 were classified as having depressive symptoms (29, 30).

### Covariates

The study covariates included individual sociodemographic characteristics and health-related factors. Sociodemographic variables included age, sex, marital status (married and others), education (elementary school and below, secondary school, and college and above), residence (rural and urban), and socioeconomic status. We defined three socioeconomic groups on the basis of tertiles of per-capita household consumption expenditure (tertile 1, 0–4283 CNY; tertile 2, 4,284–9,550 CNY; tertile 3, 9,551 CNY or more). We used annual per-capita household consumption spending as a proxy for socioeconomic status (31). Health-related factors included body mass index (BMI), self-reported smoking and drinking status (yes or no), and 14 physician-diagnosed chronic conditions (hypertension, dyslipidemia, diabetes, cancer, chronic lung diseases, liver disease, heart disease, stroke, kidney disease, digestive disease, psychiatric disease, memory-related disease, arthritis or rheumatism, and asthma). BMI was divided into three categories according to the following WHO cutoff points for Chinese: underweight (BMI < 18.5 kg/m^2^), normal weight (BMI = 18.5 to 24 kg/m^2^), and overweight or obese (BMI ≥ 24 kg/m^2^) (27). In addition, we also assessed the anemia status and cognitive function at baseline in 2015. Briefly, hemoglobin was measured at local county health centers, and the hemoglobin values of <13.0 g/dL in men and <12.0 g/dL in women are defined as anemia. Cognitive functioning was evaluated *via* a structured questionnaire in three dimensions: orientation and attention, episodic memory, and visuoconstruction. Scores on these three items were aggregated into a single score that ranged from 0 to 31, and higher scores indicate better cognitive function (32).

### Statistical Analysis

Data are presented as means ± SD or median and interquartile range for continuous variables and percentages for categorical variables. First, baseline characteristics in the cross-sectional and longitudinal analytical sample are summarized based on sarcopenia status and compared between participants using the chi-squared test, Student's *t*-test, ANOVA, Mann–Whitney *U*-test, or the Kruskal–Wallis test, as appropriate. Second, logistic regression analysis was used to estimate associations between possible sarcopenia, sarcopenia, and depressive symptoms in cross-sectional analysis. To further examine the cross-sectional associations between possible sarcopenia, sarcopenia, and specific depressive symptoms, using the previously published method (30, 33), we coded the items as dichotomous variables by defining the responses as occasionally or a moderate amount of time (3–4 days) and all of the time (5–7 days) as having the specific depressive symptoms. Logistic regression analysis was used to calculate the odds ratio (OR) with a 95% CI.

In the longitudinal analysis, we calculated the incidence rates of depressive symptoms per 1,000 person-years in CHARLS 2018. We also measured the follow-up time as the time elapsed from the date of the last interview to either the date of diagnosis of depressive symptoms or the date of the latest interview (March 2019) in which the individual participated. To examine the association between baseline sarcopenia status and incident depressive symptoms in longitudinal analysis, Cox proportional hazards models were used to calculate the hazard ratio (HR) with 95%CI. Two models were estimated: in Model 1, age, sex, residence, marital status, educational level, smoking, drinking, socioeconomic status, and BMI were included; Model 2 was adjusted as for model 1 with further adjustment for 14 chronic comorbidities, anemia, and total cognitive score. In addition, we also evaluated the association between low muscle mass alone and depressive symptoms in the cross-sectional and longitudinal analytical sample. All statistical analyses were performed retrospectively with IBM SPSS (Version 25.0, IBM Corp., Armonk, NY, USA), and R version 3.5.1 (R Foundation for Statistical Computing). In all cases, *p* < 0.05 was considered significant.

## Results

### Characteristics of Participants in the Cross-Sectional and Longitudinal Study

The baseline characteristics of the cross-sectional study participants are shown in Table 1. The mean (SD) age at baseline was 68.0 (6.5) years; 3,807 (49.4%) of the participants were men and 3,899 (50.6%) were women. Among these 7,706 older adults, the prevalence of possible sarcopenia and sarcopenia was 34.1% (2,627/7,706) and 10.0% (769/7,706), respectively. Individuals with sarcopenia were older and not married, more likely to live in rural areas, had lower socioeconomic status and educational level, and had lower BMI and total cognitive score, and higher prevalence of chronic conditions (including hypertension, chronic lung diseases, stroke, heart disease, digestive disease, memory-related disease, arthritis or rheumatism, asthma, and anemia) than the no-sarcopenia individuals (all *p* < 0.05). A comparison of characteristics between individuals not included and those who were included in the longitudinal analysis is shown in Supplementary Table 1. Supplementary Table 2 presents the baseline characteristics of 4,652 participants without depressive symptoms (46.4% women; mean age 67.4 ± 6.0) in the longitudinal study stratified by sarcopenia status. Except for gender, hypertension, digestive disease, and memory-related diseases, differences in baseline characteristics stratified by sarcopenia status were similar in the longitudinal analytical sample, as compared with the cross-sectional study.

**Table 1:** Baseline characteristics of 7,706 participants by sarcopenia status in CHARLS 2015.

**Characteristics**	**No sarcopenia (*n* = 4,310)**	**Possible sarcopenia (*n* = 2,627)**	**Sarcopenia (*n* = 769)**	***p***
Age, years	66.2 ± 5.3	69.9 ± 7.0	72.0 ± 7.2	<0.001
Female, *n* (%)	2,004 (46.5)	1,537 (58.5)	358 (46.6)	<0.001
Married (vs. others)	3,678 (85.3)	1,935 (73.7)	557 (72.4)	<0.001
Urban (vs. rural)	638 (14.8)	306 (11.6)	50 (6.5)	<0.001
Smoking^a^	1,451 (35.3)	688 (27.6)	288 (39.0)	<0.001
Drinking^a^	1,361 (37.1)	603 (26.6)	201 (30.3)	<0.001
**Educational level** ^**a**^				
Elementary school or below	2,965 (76.3)	2,047 (85.6)	630 (89.6)	<0.001
Secondary school	860 (22.1)	329 (13.8)	66 (9.4)	
College and above	63 (1.6)	16 (0.6)	7 (1.0)	
**Socioeconomic status** ^**a**^				
Tertile 1 (the poorest)	780 (29.0)	627 (37.8)	206 (41.3)	<0.001
Tertile 2	950 (35.3)	507 (30.6)	158 (31.7)	
Tertile 3 (the richest)	959 (35.7)	523 (31.6)	135 (27.1)	
**BMI category**, ***n*** **(%)**				
Underweight	244 (5.7)	140 (5.3)	223 (29.0)	<0.001
Normal weight	2,232 (51.8)	1,314 (50.0)	417 (54.2)	
Overweight or obese	1,834 (42.5)	1,173 (44.7)	129 (16.8)	
**Comorbidities**, ***n*** **(%)**				
Hypertension	1,079 (25.0)	868 (33.0)	209 (27.2)	<0.001
Dyslipidemia	472 (11.0)	334 (12.7)	54 (7.0)	<0.001
Diabetes	279 (6.5)	218 (8.3)	41 (5.3)	0.003
Cancer	42 (1.0)	30 (1.1)	12 (1.6)	0.337
Chronic lung diseases	476 (11.0)	392 (14.9)	155 (20.2)	<0.001
Liver disease	204 (4.7)	99 (3.8)	34 (4.4)	0.162
Heart disease	560 (13.0)	454 (17.3)	126 (16.4)	<0.001
Stroke	69 (1.6)	121 (4.6)	26 (3.4)	<0.001
Kidney disease	301 (7.0)	202 (7.7)	55 (7.2)	0.543
Digestive disease	1,065 (24.7)	663 (25.2)	232 (30.2)	0.006
Psychiatric disease	51 (1.2)	45 (1.7)	14 (1.8)	0.123
Memory-related disease	62 (1.4)	83 (3.2)	14 (1.8)	<0.001
Arthritis or rheumatism	1,606 (37.3)	1,177 (44.8)	336 (43.7)	<0.001
Asthma	179 (4.2)	158 (6.0)	76 (9.9)	<0.001
Anemia^a^	679 (18.6)	479 (23)	214 (35.5)	<0.001
Handgrip strength (kg)	31.2 ± 8.6	21.1 ± 8.4	20.9 ± 7.2	<0.001
ASM/Ht^2^ (kg/m^2^)	7.1 ± 1.5	7.5 ± 1.0	5.5 ± 2.1	<0.001
Total cognitive score	14 (9, 18)	10 (6, 15)	9 (5, 14)	<0.001
CES-D score	6 (3, 10)	8 (4, 14)	8 (4, 15)	<0.001
Depressive symptoms^b^	927 (21.5)	882 (33.6)	276 (35.9)	<0.001

*Data are shown as means ± SD median (interquartile range), or numbers (percentages)*.

a *Missing data: 362 for smoking, 1,103 for drinking, 722 for educational level, 2,860 for socioeconomic status, and 1,374 for anemia data*.

b *Defined as a score of 12 or greater on the 10-item Center for Epidemiologic Studies Depression Scale*.

*ASM, appendicular skeletal muscle; BMI, body mass index; CES-D, the 10-item Center for Epidemiologic Studies Depression Scale*.

### Cross-Sectional Association of Sarcopenia Status With Depressive Symptoms in CHARLS 2015

**Figure 2:**
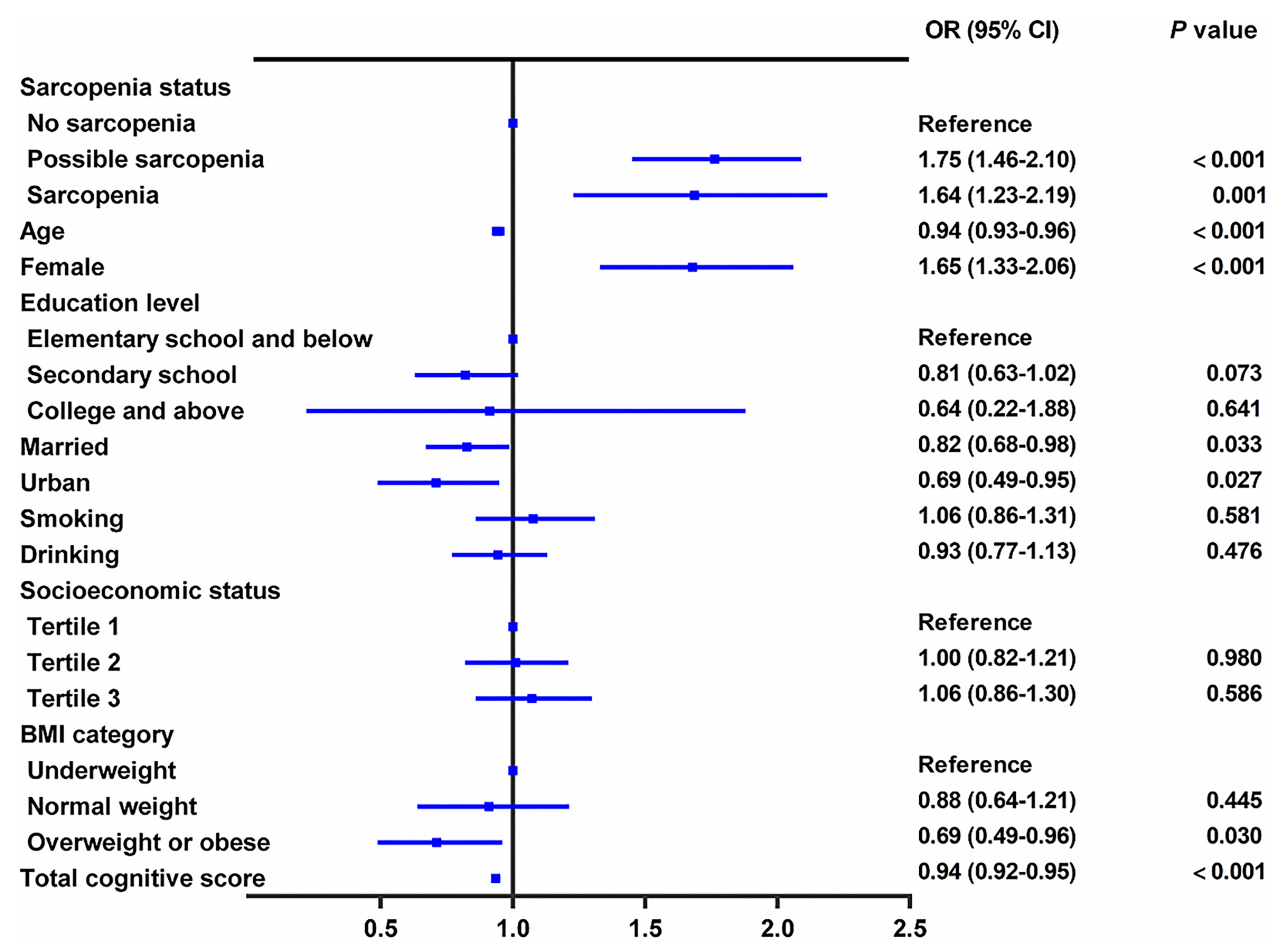
Cross-sectional association between sarcopenia status and depressive symptoms. The graph shows odds ratio (OR) and 95% CI for depressive symptoms adjusted for age, sex, residence, marital status, educational level, smoking status, drinking status, socioeconomic status, body mass index, and comorbidities, including hypertension, dyslipidemia, diabetes, cancer, chronic lung diseases, liver disease, heart disease, stroke, kidney disease, digestive disease, psychiatric disease, memory-related disease, arthritis or rheumatism, asthma, anemia, and total cognitive score.

In the cross-sectional study, persons with sarcopenia or possible sarcopenia had higher CES-D scores than no-sarcopenia individuals, and the prevalence of depressive symptoms in total populations, no-sarcopenia, possible sarcopenia, and sarcopenia individuals were 27.1% (2,085/7,706), 21.5% (927/4,310), 33.6% (882/2,627), and 35.9% (276/769), respectively (*p* for trend <0.001, Table 1). Compared with participants with no sarcopenia, both possible sarcopenia (OR: 1.75; 95% CI: 1.46–2.10, *p* < 0.001) and sarcopenia (OR: 1.64; 95% CI: 1.23–2.19, *p* = 0.001) were significantly associated with higher odds of depressive symptoms (Figure 2).

Table 2 shows the association between sarcopenia status and specific depressive symptoms after adjusting for confounders. Of the 10 individual depressive symptoms measured by the CES-D, sarcopenia was significantly associated with five symptoms, including had trouble concentrating (OR: 1.35; 95%CI: 1.01–1.79), felt depressed (OR: 1.54; 95% CI: 1.16–2.04), everything was an effort (OR: 1.88; 95% CI: 1.43–2.48), sleep was restless (OR: 1.48; 95% CI: 1.13–1.95), and could not get going (OR: 1.33; 95% CI: 1.02–1.74) (all *p* < 0.05). We also found that possible sarcopenia was significantly associated with higher odds of all the 10 individual depressive symptoms after adjusting for all covariates (all *p* < 0.01, Table 2).

**Table 2:** The odds ratio of elevated specific depressive symptoms by sarcopenia status in 2015.

**Items^**a**^**	**Symptomatic, *n***	**No-sarcopenia**	**Adjusted OR (95% CI) for possible sarcopenia**	**Adjusted OR (95% CI) for sarcopenia**
Bothered by little things	716 (15.4)	Reference	1.51 (1.26, 1.80)^***^	1.32 (0.99, 1.77)^+^
Had trouble concentrating	832 (17.9)	Reference	1.35 (1.13, 1.61)^**^	1.35 (1.01, 1.79)^*^
Felt depressed	842 (18.1)	Reference	1.49 (1.25, 1.78)^***^	1.54 (1.16, 2.04)^**^
Everything was an effort	920 (19.8)	Reference	1.55 (1.30, 1.85)^***^	1.88 (1.43, 2.48)^***^
Did not feel hopeful	983 (21.1)	Reference	1.25 (1.06, 1.47)^**^	1.14 (0.87, 1.49)
Felt fearful	357 (7.7)	Reference	1.97 (1.51, 2.57)^***^	1.37 (0.90, 2.09)
Sleep was restless	825 (17.7)	Reference	1.35 (1.14, 1.60)^**^	1.48 (1.13, 1.95)^**^
Did not feel happy	1,158 (24.9)	Reference	1.52 (1.25, 1.85)^***^	1.16 (0.84, 1.58)
Felt lonely	659 (14.2)	Reference	1.50 (1.18, 1.90)^**^	0.99 (0.68, 1.45)
Could not get going	355 (7.6)	Reference	1.39 (1.17, 1.64)^***^	1.33 (1.02, 1.74)^*^

a *Measured by the 10-item Center for Epidemiologic Studies Depression Scale. Model was adjusted for age, sex, residence, marital status, educational level, smoking status, drinking status, socioeconomic status, body mass index, all comorbidities (hypertension, dyslipidemia, diabetes, cancer, chronic lung diseases, liver disease, heart disease, stroke, kidney disease, digestive disease, psychiatric disease, memory-related disease, arthritis or rheumatism, asthma), anemia, and total cognitive score*.

+ *p < 0.10*.

* *p < 0.05*.

** *p < 0.01*.

*** *p < 0.001*.

### Longitudinal Association Between Baseline Sarcopenia Status and Incident Depressive Symptoms at Follow-Up, 2015–2018

**Figure 3:**
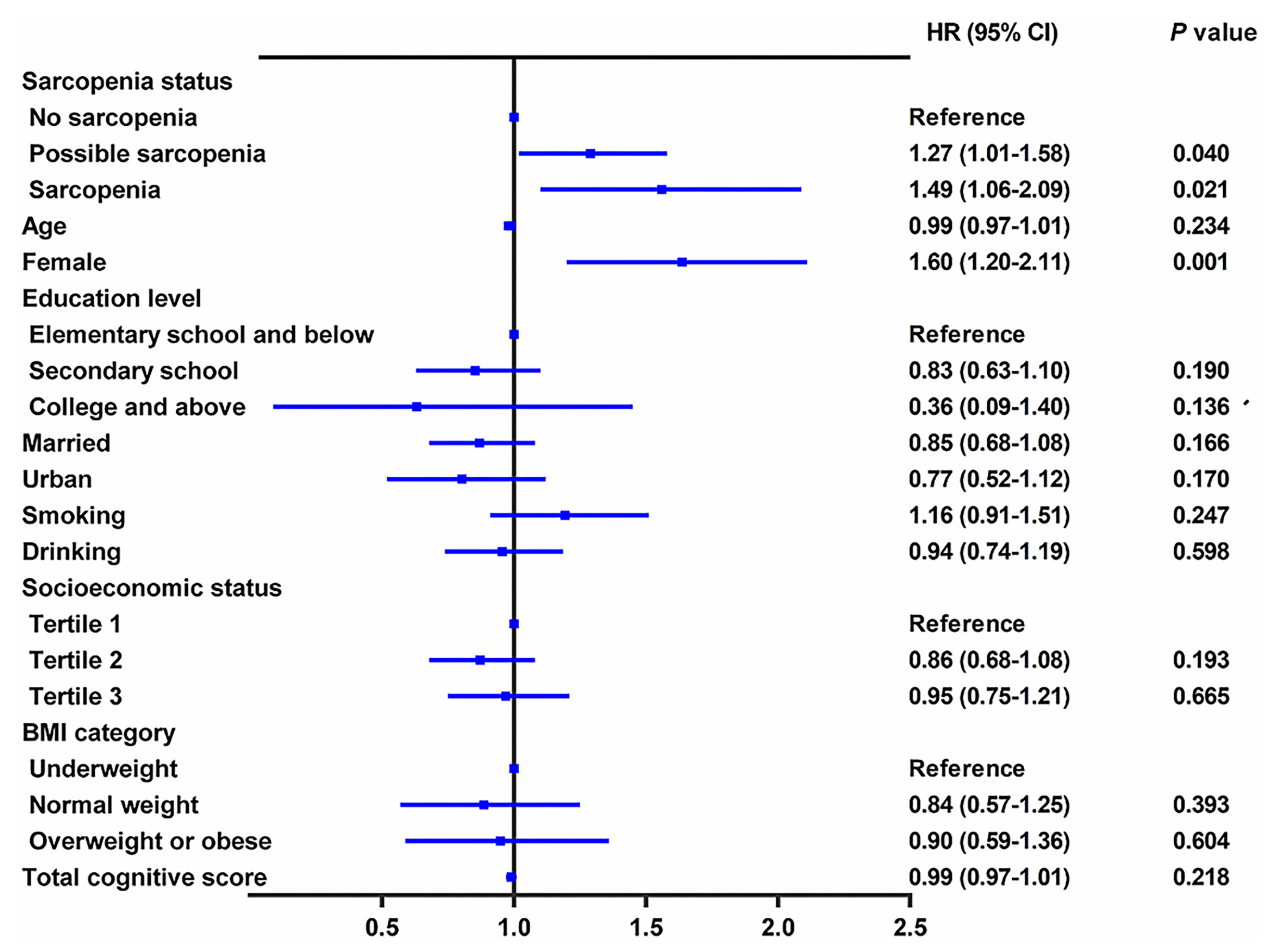
Longitudinal association of baseline sarcopenia status with incident depressive symptoms, 2015–2018. The graph shows hazard ratio (HR) and 95% CI for depressive symptoms risk adjusted for age, sex, residence, marital status, educational level, smoking status, drinking status, socioeconomic status, body mass index, all comorbidities, anemia, and total cognitive score.

During the follow-up period between CHARLS 2015 and CHARLS 2018, 956 cases (20.6%) with incident depressive symptoms were identified (Table 3). During the 3.7 years of follow-up, the incidence rate of depressive symptoms was 47.09 per 1,000 person-years among participants with no-sarcopenia, 69.56 per 1,000 person-years among possible sarcopenia, and 69.62 per 1,000 person-years among participants with sarcopenia. Table 3 shows the relation between baseline sarcopenia status and incident depressive symptoms in the longitudinal analysis. After adjusting for age, sex, residence, marital status, educational level, smoking status, drinking status, socioeconomic status, and BMI (in model 1), the presence of sarcopenia was independently associated with a 53.0% increased risk of depressive symptoms (adjusted HR:1.53; 95% CI: 1.12–2.09, *p* < 0.01). Individuals with possible sarcopenia had a 44.0% increased risk of incident depressive symptoms (adjusted HR: 1.44; 95% CI: 1.18–1.76, *p* = 0.008). The results did not significantly change after further adjusting for comorbidities, anemia, and total cognitive score (in model 2). Individuals with the diagnosed possible sarcopenia (adjusted HR: 1.27; 95% CI: 1.01–1.58, *p* = 0.040) and sarcopenia participants (adjusted HR: 1.49; 95% CI: 1.06–2.09, *p* = 0.021) were more likely to have incident depressive symptoms than no-sarcopenia peers (Table 3 and Figure 3).

**Table 3:** Incidence of depressive symptoms according to baseline sarcopenia status, 2015–2018.

**Sarcopenia status**	**Cases, no**.	**Incidence rate, per 1,000 person-years**	**HR (95% CI)**	
			**Model 1^**a**^**	**Model 2^**b**^**
No sarcopenia	531	47.09	Reference	Reference
Possible sarcopenia	326	69.56	1.44 (1.18, 1.76)^***^	1.27 (1.01, 1.58)^*^
Sarcopenia	99	69.62	1.53 (1.12, 2.09)^**^	1.49 (1.06, 2.09)^*^

*HR, hazard ratio*.

a *Model 1 was adjusted for age, sex, residence, marital status, educational level, smoking status, drinking status, socioeconomic status, and body mass index*.

b *Model 2 was adjusted as for model 1 with further adjustment for comorbidities (hypertension, dyslipidemia, diabetes, cancer, chronic lung diseases, liver disease, heart disease, stroke, kidney disease, digestive disease, psychiatric disease, memory-related disease, arthritis, or rheumatism, asthma), anemia, and total cognitive score*.

* *p < 0.05*.

** *p < 0.01*.

*** *p < 0.001*.

### Cross-Sectional and Longitudinal Associations of Low Muscle Mass Alone With Depressive Symptoms

In the cross-sectional analysis, a total of 4,310 individuals (46.5% women; mean age 66.2 ± 5.3) were included, and 767 individuals (17.8%) had low muscle mass alone with neither low handgrip strength nor low physical performance. The prevalence of depressive symptoms was 20.8% (738/3,543) among individuals without any sarcopenia components and 24.6% (189/767) among individuals with low muscle mass alone (Supplementary Table 3). Individuals with low muscle mass alone were not significantly associated with higher odds of depressive symptoms (OR: 0.94; 95% CI: 0.69–1.29, *p* = 0.700). In the longitudinal analysis, a total of 2,949 participants (56.3% women; mean age 66.0 ± 5.1) were included, and 494 participants (16.8%) had low muscle mass alone with neither low handgrip strength nor low physical performance. The incidence rate of depressive symptoms was 44.77 per 1,000 person-years among participants without any sarcopenia components and 58.06 per 1,000 person-years among participants with low muscle mass alone (Table 4). Compared with the individuals without any sarcopenia components, those having low muscle mass alone in the absence of low grip strength and low physical performance were not significantly associated with an increased risk of incident depressive symptoms after adjusting for potential confounders (HR: 1.20; 95% CI: 0.85–1.69, *p* = 0.311).

**Table 4:** Longitudinal association of low muscle mass alone with incident depressive symptoms, 2015–2018.

	**Cases, no**.	**Incidence rate, per 1,000 person-years**	**HR (95% CI)**	
			**Model 1^**a**^**	**Model 2^**b**^**
**Muscle mass (*****n*** **= 2,949)**				
Reference (*n* = 2,455)	411	44.77	1	1
Low muscle mass alone (*n* = 494)	107	58.06	1.54 (0.83, 1.60)	1.20 (0.85, 1.69)

*HR, hazard ratio. Reference was a group of participants without any sarcopenia components. Low muscle mass alone, low muscle mass with neither low grip strength nor slow physical performance*.

a *Model 1 was adjusted for age, sex, residence, marital status, educational level, smoking status, drinking status, socioeconomic status, and body mass index*.

b *Model 2 was adjusted as model 1 with further adjustment for comorbidities, anemia, and total cognitive score*.

## Discussion

To our knowledge, using a large nationally representative survey of the Chinese population, this study is the first attempt to investigate the cross-sectional associations between possible sarcopenia, sarcopenia, and depressive symptoms, and the effect of baseline sarcopenia status on the risk of subsequent depressive symptoms among Chinese older adults. We found that both possible sarcopenia and sarcopenia, assessed using the AWGS 2019 algorithm, were independently and positively associated with higher odds of depressive symptoms in the cross-sectional analysis. Importantly, individuals with diagnosed possible sarcopenia or sarcopenia were at higher risk of incident depressive symptoms among Chinese older adults. There was no significant increased risk of depressive symptoms for participants with low muscle mass alone in the absence of low grip strength and low physical performance.

In the cross-sectional study, we found that sarcopenia was independently associated with higher odds of depressive symptoms, and individuals with sarcopenia were more likely to report a higher CES-D score than no-sarcopenia peers. These findings are in line with several cross-sectional studies (16–18, 20, 21), suggesting that the presence of sarcopenia was closely correlated with higher odds of depressive symptoms and/or depression. A meta-analysis comprising of 15 observational studies conducted by Chang et al. (17) showed that sarcopenia demonstrated a significant positive association with depression after adjusting for potential confounders. Wang et al. (18), whose study focused on the elderly Asian population, demonstrated that sarcopenia was strongly associated with depressive symptoms among 865 elderly Chinese individuals. Moreover, the ELSA-Brasil Study (34) suggested that depression was associated with sarcopenia as defined by the National Institutes of Health criteria among 5,927 middle-aged and older Brazilian adults, and this association was mainly due to low muscle strength. However, there is a paucity of research on the causal relationship between sarcopenia and depressive symptoms in Asia. To our knowledge, this is the first large, population-based longitudinal study that has found sarcopenia is independently associated with an increased incidence of depressive symptoms among Chinese older adults. We found that individuals with sarcopenia had a 49% higher risk of new onset depressive symptoms compared with the no-sarcopenia group after the adjustment for the potential confounders. These results may indicate that assessment of sarcopenia in older adults might facilitate identification of those at greatest risk of incident depressive symptoms, who would benefit most from early intervention. The underlying mechanisms of the association between sarcopenia and depressive symptoms are multifactorial, involving common molecule-driven pathways (35, 36), including neurotrophins, chronic inflammation, and oxidative stress, and similar lifestyle factors (1, 2), such as malnutrition and physical inactivity. Exercise has been shown to ameliorate the process of sarcopenia and depression (1, 2, 37). Mounting evidence indicates that physical activity can diminish the blood-brain barrier permeability and function as it reinforces antioxidative capacity, reduce oxidative stress, alter neurotrophic factor status, and have anti-inflammatory effects (38–40). In particular, the muscle–brain endocrine loop mediated by myokine is also involved in the associations between sarcopenia and several health problems, such as depression, anxiety, and cognitive decline (40–43). In general, skeletal muscle can produce and secrete several cytokines and peptides, namely myokines that improve brain functions, including mood, cognitive, and neuronal injury protection, showing the existence of muscle–brain crosstalk (41, 42).

Based on the AWGS 2019 criteria, the diagnostic algorithm for sarcopenia consists of three components, namely muscle strength, skeletal muscle mass, and physical performance. The AWGS 2019 also recommended a new entity of “possible sarcopenia” to facilitate early lifestyle intervention in prevention settings (5). In addition to sarcopenia, the current study also demonstrated interesting findings regarding the effect of possible sarcopenia and low muscle mass alone on depressive symptoms. Compared with participants with no sarcopenia, possible sarcopenia was positively associated with higher odds of depressive symptoms in the cross-sectional analysis. As part of the results, we also found that participants with low muscle mass alone were not significantly associated with depressive symptoms. Consistent with our findings, the Toyota Prevention Intervention for Cognitive Decline and Sarcopenia study (20) reported that both low physical performance and low muscle strength were significantly associated with depressive mood among 432 Japanese older adults, but decreased muscle mass was not. In addition, the ELSA-Brasil Study also reported that depression was not associated with low muscle mass in the cross-sectional study with 5,927 middle-aged and older Brazilian adults (34). Furthermore, several studies have proven that low grip strength, a component of possible sarcopenia, was independently associated with depression (34, 44). In the current study, we tried to confirm the causal relationships between possible sarcopenia, low muscle mass alone, and depressive symptoms. In the longitudinal analysis, we first found that individuals with the diagnosed possible sarcopenia were associated with a significantly increased risk of incident depressive symptoms. But another component of sarcopenia, low muscle mass alone, did not show an increased risk of incident depressive symptoms. Our findings support the validity of current major criteria of possible sarcopenia by the AWGS 2019, and it also suggested that maintaining enough muscle strength and/or physical performance could be beneficial in the prevention of depressive symptoms for older adults. At the same time, early diagnosis, lifestyle interventions, and prevention of possible sarcopenia in routine clinical practice should be taken as a factor in fighting against depressive symptoms and promoting healthy aging.

Plenty of previous studies (16, 18, 20, 21) on the relationship of sarcopenia with depressive symptoms only regarded the presence of depressive symptoms as a binary variable. However, this schema discards data about specific symptom items, treating all as equivalent and interchangeable indicators of depressive symptoms (45). Moreover, two sarcopenic persons with equal CES-D scores may have different severity of clinical symptoms. To date, no studies have assessed the association between sarcopenia status and specific depressive symptoms of CES-D-10 among Chinese older adults. In this study, we found that sarcopenia was significantly associated with certain depressive symptoms, such as having trouble concentrating, feeling depressed, everything was an effort, sleep was restless, and could not get going. Our findings are consistent with evidence from the ELSA-Brasil Study (34) showing that several depressive symptom items, such as impaired concentration, sleep problems, and depressed mood, were associated with sarcopenia among middle-aged and older Brazilian adults. Our results suggest that elderly people who are at high risk of sarcopenia should be screened and intervened for specific depressive symptoms items.

There are several strengths to this study. First, we used a large and nationally representative sample, thereby allowing for broad generalizability of our findings to the Chinese older population. Second, unlike most previous cross-sectional studies, this is the first study to explore the longitudinal relationships between possible sarcopenia, sarcopenia, and depressive symptoms in Asia. In addition, the study was also the first one to examine the associations between sarcopenia status and specific depressive symptoms items of CES-D-10 among Chinese older adults. More importantly, our findings supported the validity of the current major algorithm of possible sarcopenia by the AWGS 2019 and suggested that preventing and/or improving both possible sarcopenia and sarcopenia may be beneficial in fighting against depressive symptoms and promoting healthy aging for Chinese older adults.

However, there are limitations to this study. First, in our study, individuals walked a 2.5-m course at their normal pace two times, rather than a 6-m walk. However, a systematic review comprising of 48 studies found that the “distance walked did not influence the recorded gait speed” among older adults (46). Moreover, Wu et al. (27) reported that the average gait speed among individuals with sarcopenia and without sarcopenia in CHARLS 2015 was consistent with the results of a previous Chinese study. Therefore, a 2.5-m walk might be appropriate for assessing the walking speed of Chinese older residents. Second, the information about the SPPB score are completely missing, so the estimates may have some degree of bias. Third, this study used observational data, which may have biased the observed relations by introducing confounding factors. To reduce such bias, we considered as many related factors as possible in the analysis; however, other potential confounding factors, such as nutritional status, hypoalbuminemia, food habits, physical inactivity, and sleep behavior, cannot be ruled out. Despite these limitations, our findings will provide essential clues for future research. Future studies are needed to pinpoint the role of depressive symptoms in the new onset of sarcopenia. With respect to the clinical implications of our findings, integration of both sarcopenia and depressive symptoms assessment should be introduced to community-based health checkups and routine clinical practice of older adults.

## Conclusion

In conclusion, both possible sarcopenia and sarcopenia, assessed using the AWGS 2019 criteria, were independent predictors for the occurrence of depressive symptoms among Chinese older adults. Our findings provided new evidence supporting the longitudinal connection between sarcopenia and mental health problems in the context of aging, it also provides further justification for timely identification and management of both possible sarcopenia and sarcopenia as part of comprehensive strategies to fight against depressive symptoms in Chinese community-dwelling older adults.

## Data Availability Statement

The original contributions presented in the study are included in the article/Supplementary Material, further inquiries can be directed to the corresponding authors.

## Ethics Statement

The studies involving human participants were reviewed and approved by the protocol was approved by the Ethical Review Committee of Peking University (Approval No. IRB00001052-11015). The patients/participants provided their written informed consent to participate in this study.

## Author Contributions

DZ and KG conceived the protocol. KG, DZ, TL, W-ZM, SH, B-LL, LZ, and JZ contributed to the analysis and interpretation of data. KG and DZ grafted the manuscript. W-ZM, TL, and SH critically revised the manuscript. All authors agree to be fully accountable for ensuring the integrity and accuracy of the work and read and approved the final manuscript.

## Conflict of Interest

The authors declare that the research was conducted in the absence of any commercial or financial relationships that could be construed as a potential conflict of interest.

## Publisher's Note

All claims expressed in this article are solely those of the authors and do not necessarily represent those of their affiliated organizations, or those of the publisher, the editors and the reviewers. Any product that may be evaluated in this article, or claim that may be made by its manufacturer, is not guaranteed or endorsed by the publisher.

## References

[1] Cruz-Jentoft AJ Sayer AA. Sarcopenia. Lancet. (2019) 393:2636–46. 10.1016/S0140-6736(19)31138-9 31171417

[2] Bauer J Morley JE Schols A Ferrucci L Cruz-Jentoft AJ Dent E. Sarcopenia: a time for Action. An SCWD position paper. J Cachexia Sarcopenia Muscle. (2019) 10:956–61. 10.1002/jcsm.12483 31523937 PMC6818450

[3] Cruz-Jentoft AJ Baeyens JP Bauer JM Boirie Y Cederholm T Landi F. Sarcopenia: European consensus on definition and diagnosis: report of the European working group on sarcopenia in older people. Age Ageing. (2010) 39:412–23. 10.1093/ageing/afq034 20392703 PMC2886201

[4] Chen LK Liu LK Woo J Assantachai P Auyeung TW Bahyah KS. Sarcopenia in Asia: consensus report of the Asian working group for sarcopenia. J Am Med Dir Assoc. (2014) 15:95–101. 10.1016/j.jamda.2013.11.025 24461239

[5] Chen LK Woo J Assantachai P Auyeung TW Chou MY Iijima K. Asian working group for sarcopenia: 2019 consensus update on sarcopenia diagnosis and treatment. J Am Med Dir Assoc. (2020) 21:300–7. 10.1016/j.jamda.2019.12.012 32033882

[6] Cruz-Jentoft AJ Bahat G Bauer J Boirie Y Bruyère O Cederholm T. Sarcopenia: revised European consensus on definition and diagnosis. Age Ageing. (2019) 48:16–31. 10.1093/ageing/afz046 30312372 PMC6322506

[7] Cawthon PM Lui LY Taylor BC McCulloch CE Cauley JA Lapidus J. Clinical definitions of sarcopenia and risk of hospitalization in community-dwelling older men: the osteoporotic fractures in men study. J Gerontol A Biol Sci Med Sci. (2017) 72:1383–9. 10.1093/gerona/glw327 28329087 PMC5861971

[8] Kitamura A Seino S Abe T Nofuji Y Yokoyama Y Amano H. Sarcopenia: prevalence, associated factors, and the risk of mortality and disability in Japanese older adults. J Cachexia Sarcopenia Muscle. (2021) 12:30–8. 10.1002/jcsm.12651 33241660 PMC7890144

[9] Zhang X Huang P Dou Q Wang C Zhang W Yang Y. Falls among older adults with sarcopenia dwelling in nursing home or community: a meta-analysis. Clin Nutr. (2020) 39:33–9. 10.1016/j.clnu.2019.01.002 30665817

[10] Sayer AA. Sarcopenia. Bmj. (2010) 341:c4097. 10.1136/bmj.c4097 20699307

[11] Whiteford HA Degenhardt L Rehm J Baxter AJ Ferrari AJ Erskine HE. Global burden of disease attributable to mental and substance use disorders: findings from the global burden of disease study 2010. Lancet. (2013) 382:1575–86. 10.1016/S0140-6736(13)61611-6 23993280

[12] Malhi GS Mann JJ. Depression. Lancet. (2018) 392:2299–312. 10.1016/S0140-6736(18)31948-2 30396512

[13] Bhattacharya R Shen C Sambamoorthi U. Excess risk of chronic physical conditions associated with depression and anxiety. BMC Psychiatry. (2014) 14:10. 10.1186/1471-244X-14-10 24433257 PMC3902063

[14] Kozela M Bobak M Besala A Micek A Kubinova R Malyutina S. The association of depressive symptoms with cardiovascular and all-cause mortality in central and Eastern Europe: prospective results of the HAPIEE study. Eur J Prev Cardiol. (2016) 23:1839–47. 10.1177/2047487316649493 27154591 PMC5089224

[15] Kim NH Kim HS Eun CR Seo JA Cho HJ Kim SG. Depression is associated with sarcopenia, not central obesity, in elderly Korean men. J Am Geriatr Soc. (2011) 59:2062–8. 10.1111/j.1532-5415.2011.03664.x 22092258

[16] Hsu YH Liang CK Chou MY Liao MC Lin YT Chen LK. Association of cognitive impairment, depressive symptoms and sarcopenia among healthy older men in the veterans retirement community in southern Taiwan: a cross-sectional study. Geriatr Gerontol Int. (2014) 14 Suppl 1:102–8. 10.1111/ggi.12221 24450567

[17] Chang KV Hsu TH Wu WT Huang KC Han DS. Is sarcopenia associated with depression? A systematic review and meta-analysis of observational studies. Age Ageing. (2017) 46:738–46. 10.1093/ageing/afx094 28633395

[18] Wang H Hai S Liu Y Cao L Liu Y Liu P. Association between depressive symptoms and sarcopenia in older Chinese community-dwelling individuals. Clin Interv Aging. (2018) 13:1605–11. 10.2147/CIA.S173146 30233157 PMC6130547

[19] Wang Q Tian W. Prevalence, awareness, and treatment of depressive symptoms among the middle-aged and elderly in China from 2008 to 2015. Int J Health Plann Manage. (2018) 33:1060–70. 10.1002/hpm.2581 30074651

[20] Hayashi T Umegaki H Makino T Cheng XW Shimada H Kuzuya M. Association between sarcopenia and depressive mood in urban-dwelling older adults: a cross-sectional study. Geriatr Gerontol Int. (2019) 19:508–12. 10.1111/ggi.13650 30884107

[21] Kilavuz A Meseri R Savas S Simsek H Sahin S Bicakli DH. Association of sarcopenia with depressive symptoms and functional status among ambulatory community-dwelling elderly. Arch Gerontol Geriatr. (2018) 76:196–201. 10.1016/j.archger.2018.03.003 29550658

[22] Kim JK Choi SR Choi MJ Kim SG Lee YK Noh JW. Prevalence of and factors associated with sarcopenia in elderly patients with end-stage renal disease. Clin Nutr. (2014) 33:64–8. 10.1016/j.clnu.2013.04.002 23631844

[23] Zhao Y Hu Y Smith JP Strauss J Yang G. Cohort profile: the China health and retirement longitudinal study (CHARLS). Int J Epidemiol. (2014) 43:61–8. 10.1093/ije/dys203 23243115 PMC3937970

[24] Wen X Wang M Jiang CM Zhang YM. Anthropometric equation for estimation of appendicular skeletal muscle mass in Chinese adults. Asia Pac J Clin Nutr. (2011) 20:551–6. 22094840

[25] Yang M Hu X Wang H Zhang L Hao Q Dong B. Sarcopenia predicts readmission and mortality in elderly patients in acute care wards: a prospective study. J Cachexia Sarcopenia Muscle. (2017) 8:251–8. 10.1002/jcsm.12163 27896949 PMC5377397

[26] Alexandre TdaSDuarteYASantosJLWongRLebrãoML. Sarcopenia according to the European working group on sarcopenia in older people (EWGSOP) versus dynapenia as a risk factor for mortality in the elderly. J Nutr Health Aging. (2014) 18:751–6. 10.1007/s12603-014-0540-225286455

[27] Wu X Li X Xu M Zhang Z He L Li Y. Sarcopenia prevalence and associated factors among older Chinese population: findings from the China health and retirement longitudinal study. PLoS ONE. (2021) 16:e0247617. 10.1371/journal.pone.0247617 33661964 PMC7932529

[28] Andresen EM Malmgren JA Carter WB Patrick DL. Screening for depression in well older adults: evaluation of a short form of the CES-D (center for epidemiologic studies depression scale). Am J Prev Med. (1994) 10:77–84. 10.1016/S0749-3797(18)30622-6 8037935

[29] Chen H Mui AC. Factorial validity of the Center for Epidemiologic Studies Depression scale short form in older population in China. Int Psychogeriatr. (2014) 26:49–57. 10.1017/S1041610213001701 24125553

[30] Li H Zheng D Li Z Wu Z Feng W Cao X. Association of depressive symptoms with incident cardiovascular diseases in middle-aged and older Chinese adults. JAMA Netw Open. (2019) 2:e1916591. 10.1001/jamanetworkopen.2019.16591 31800066 PMC6902756

[31] Zhao Y Atun R Oldenburg B McPake B Tang S Mercer SW. Physical multimorbidity, health service use, and catastrophic health expenditure by socioeconomic groups in China: an analysis of population-based panel data. Lancet Glob Health. (2020) 8:e840–9. 10.1016/S2214-109X(20)30127-3 32446349 PMC7241981

[32] Cao L Zhao Z Ji C Xia Y. Association between solid fuel use and cognitive impairment: a cross-sectional and follow-up study in a middle-aged and older Chinese population. Environ Int. (2021) 146:106251. 10.1016/j.envint.2020.106251 33248346

[33] Jokela M Virtanen M Batty GD Kivimäki M. Inflammation and specific symptoms of depression. JAMA Psychiatry. (2016) 73:87–8. 10.1001/jamapsychiatry.2015.1977 26579988

[34] Szlejf C Suemoto CK Brunoni AR Viana MC Moreno AB Matos SMA. Depression is associated with sarcopenia due to low muscle strength: results from the ELSA-Brasil study. J Am Med Dir Assoc. (2019) 20:1641–6. 10.1016/j.jamda.2018.09.020 30409492

[35] Schaap LA Pluijm SM Deeg DJ Harris TB Kritchevsky SB Newman AB. Higher inflammatory marker levels in older persons: associations with 5-year change in muscle mass and muscle strength. J Gerontol A Biol Sci Med Sci. (2009) 64:1183–9. 10.1093/gerona/glp097 19622801 PMC2759573

[36] Berk M Williams LJ Jacka FN O'Neil A Pasco JA Moylan S. So depression is an inflammatory disease, but where does the inflammation come from? BMC Med. (2013) 11:200. 10.1186/1741-7015-11-200 24228900 PMC3846682

[37] Agudelo LZ Femenía T Orhan F Porsmyr-Palmertz M Goiny M Martinez-Redondo V. Skeletal muscle PGC-1α1 modulates kynurenine metabolism and mediates resilience to stress-induced depression. Cell. (2014) 159:33–45. 10.1016/j.cell.2014.07.051 25259918

[38] Małkiewicz MA Szarmach A Sabisz A Cubała WJ Szurowska E Winklewski PJ. Blood-brain barrier permeability and physical exercise. J Neuroinflammation. (2019) 16:15. 10.1186/s12974-019-1403-x 30678702 PMC6345022

[39] Mokhtarzade M Motl R Negaresh R Zimmer P Khodadoost M Baker JS. Exercise-induced changes in neurotrophic factors and markers of blood-brain barrier permeability are moderated by weight status in multiple sclerosis. Neuropeptides. (2018) 70:93–100. 10.1016/j.npep.2018.05.010 29880392

[40] Chen W Wang L You W Shan T. Myokines mediate the cross talk between skeletal muscle and other organs. J Cell Physiol. (2021) 236:2393–412. 10.1002/jcp.30033 32885426

[41] Pedersen BK. Physical activity and muscle-brain crosstalk. Nat Rev Endocrinol. (2019) 15:383–92. 10.1038/s41574-019-0174-x 30837717

[42] Scisciola L Fontanella RA Surina Cataldo V Paolisso G Barbieri M. Sarcopenia and cognitive function: role of myokines in muscle brain cross-talk. Life (Basel). (2021) 11:173. 10.3390/life11020173 33672427 PMC7926334

[43] Severinsen MCK Pedersen BK. Muscle-organ crosstalk: the emerging roles of myokines. Endocr Rev. (2020) 41:594–609. 10.1210/endrev/bnaa016 32393961 PMC7288608

[44] Brooks JM Titus AJ Bruce ML Orzechowski NM Mackenzie TA Bartels SJ. Depression and handgrip strength among U.S. adults aged 60 years and older from NHANES 2011–2014. J Nutr Health Aging. (2018) 22:938–43. 10.1007/s12603-018-1041-5 30272097 PMC6168750

[45] Fried EI Nesse RM. Depression sum-scores don't add up: why analyzing specific depression symptoms is essential. BMC Med. (2015) 13:72. 10.1186/s12916-015-0325-4 25879936 PMC4386095

[46] Peel NM Kuys SS Klein K. Gait speed as a measure in geriatric assessment in clinical settings: a systematic review. J Gerontol A Biol Sci Med Sci. (2013) 68:39–46. 10.1093/gerona/gls174 22923430

