# Supplementary material for: Association Between Sarcopenia and Depressive Symptoms in Chinese Older Adults: Evidence From the China Health and Retirement Longitudinal Study
Source: Front Med (Lausanne). 2021 Nov 17;8:755705. doi: 10.3389/fmed.2021.755705 (PMC8635632; doi:10.3389/fmed.2021.755705)
Supplement: Supplementary file 1 [file Table_1.DOCX]

**Association between sarcopenia and depressive symptoms in Chinese older adults: Evidence from the China Health and Retirement Longitudinal Study**

Ke Gao, Wen-Zhuo Ma, Scarlett Huck, Bo-Lin Li, Li Zhang, Jiao Zhu, Tian Li**^*^**, Dan Zhou**^*^**

**Supplementary Table 1 Baseline characteristics between participants not included and included in the longitudinal analysis**

| **Characteristics** | \| **Excluded** \| \| --- \|   **(n=3054)** | **Included**  **(n=4652)** | ***P*** |
| --- | --- | --- | --- | --- |
| Age, y | 69.1 ± 6.9 | 67.4 ± 6.0 | *<*0.001 |
| Female, n (%) | 1742 (57.0) | 2157 (46.4) | *<*0.001 |
| Married (vs others) | 2264 (74.1) | 3906 (84.0) | *<*0.001 |
| Urban (vs rural) | 326 (10.7) | 668 (14.4) | *<*0.001 |
| Smoking^a^ | 881 (30.3) | 1546 (34.9) | *<*0.001 |
| Drinking^a^ | 754 (28.7) | 1411 (35.5) | *<*0.001 |
| Educational level^a^ |  |  | *<*0.001 |
| Elementary school or below | 2404 (86.5) | 3238 (77.0) |  |
| Secondary school | 352 (12.7) | 903 (21.5) |  |
| College and above | 24 (0.9) | 62 (1.5) |  |
| Socioeconomic status^a^ |  |  | 0.002 |
| Tertile 1 (the poorest) | 713 (36.2) | 900 (31.3) |  |
| Tertile 2 | 626 (31.8) | 989 (34.4) |  |
| Tertile 3 (the richest) | 632 (32.1) | 985 (34.3) |  |
| BMI category, n (%) |  |  | *<*0.001 |
| Underweight | 315 (10.3) | 292 (6.3) |  |
| Normal weight | 1603 (52.5) | 2360 (50.7) |  |
| Overweight or obese | 1136 (37.2) | 2000 (43.0) |  |
| Comorbidities, n (%) |  |  |  |
| Hypertension | 933 (30.6) | 1223 (26.3) | *<*0.001 |
| Dyslipidemia | 346 (11.3) | 514 (11.0) | 0.702 |
| Diabetes | 258 (8.4) | 280 (6.0) | *<*0.001 |
| Cancer | 40 (1.3) | 44 (0.9) | 0.132 |
| Chronic lung diseases | 495 (16.2) | 528 (11.3) | *<*0.001 |
| Liver disease | 145 (4.7) | 192 (4.1) | 0.193 |
| Heart disease | 530 (17.4) | 610 (13.1) | *<*0.001 |
| Stroke | 107 (3.5) | 109 (2.3) | 0.003 |
| Kidney disease | 285 (9.3) | 273 (5.9) | *<*0.001 |
| Digestive disease | 876 (28.7) | 1084 (23.3) | *<*0.001 |
| Psychiatric disease | 59 (1.9) | 51 (1.1) | 0.002 |
| Memory-related disease | 95 (3.1) | 64 (1.4) | *<*0.001 |
| Arthritis or rheumatism | 1441 (47.2) | 1678 (36.1) | *<*0.001 |
| Asthma | 209 (6.8) | 204 (4.4) | *<*0.001 |
| Anaemia^a^ | 621 (25.7) | 751 (19.2) | *<*0.001 |
| Handgrip strength, kg | 24.3 ± 9.9 | 28.3 ± 9.4 | *<*0.001 |
| ASM/Ht^2^, kg/m^2^ | 7.0 ± 1.6 | 7.2 ± 1.5 | *<*0.001 |
| Total cognitive score | 10 (5, 15) | 14 (9, 17) | *<*0.001 |

Data are shown as means ± standard deviation, median (interquartile range), or numbers (percentages).

^a^ Missing data: 362 for smoking, 1103 for drinking, 722 for educational level, 2860 for socioeconomic status, and 1374 for anaemia data.

Abbreviation: ASM, appendicular skeletal muscle; BMI, body mass index.

**Supplementary Table 2. Baseline characteristics of 4652 participants without depressive symptoms by sarcopenia status in 2015.**

| **Characteristics** | **No sarcopenia (n=3017)** | **Possible sarcopenia (n=1255)** | **Sarcopenia**  **(n=380)** | ***P*** |
| --- | --- | --- | --- | --- |
| Age, y | 66.1 ± 5.2 | 69.2 ± 6.6 | 71.4 ± 7.1 | *<*0.001 |
| Female, n (%) | 1329 (44.1) | 674 (53.7) | 154 (40.5) | *<*0.001 |
| Married (vs others) | 2637 (87.4) | 971 (77.4) | 298 (78.4) | *<*0.001 |
| Urban (vs rural) | 475 (15.7) | 163 (13.0) | 30 (7.9) | *<*0.001 |
| Smoking^a^ | 1048 (36.3) | 341 (28.7) | 157 (43.4) | *<*0.001 |
| Drinking^a^ | 997 (38.6) | 305 (28.5) | 109 (34.0) | *<*0.001 |
| Educational level^a^ |  |  |  | *<*0.001 |
| Elementary school or below | 2008 (73.6) | 932 (82.0) | 298 (87.6) |  |
| Secondary school | 672 (24.6) | 194 (17.1) | 37 (10.9) |  |
| College and above | 47 (1.7) | 10 (0.9) | 5 (1.5) |  |
| Socioeconomic status^a^ |  |  |  | *<*0.001 |
| Tertile 1 (the poorest) | 562 (30.4) | 291 (37.5) | 103 (41.2) |  |
| Tertile 2 | 644 (34.8) | 233 (30.0) | 81 (32.4) |  |
| Tertile 3 (the richest) | 642 (34.7) | 252 (32.5) | 66 (26.4) |  |
| BMI category, n (%) |  |  |  | *<*0.001 |
| Underweight | 147 (4.9) | 43 (3.4) | 102 (26.8) |  |
| Normal weight | 1535 (50.9) | 600 (47.8) | 225 (59.2) |  |
| Overweight or obese | 1335 (44.2) | 612 (48.8) | 53 (13.9) |  |
| Comorbidities, n (%) |  |  |  |  |
| Hypertension | 734 (24.3) | 399 (31.8) | 90 (23.7) | *<*0.001 |
| Dyslipidemia | 329 (10.9) | 158 (12.6) | 27 (7.1) | 0.011 |
| Diabetes | 162 (5.4) | 97 (7.7) | 21 (5.5) | 0.012 |
| Cancer | 27 (0.9) | 12 (1.0) | 5 (1.3) | 0.726 |
| Chronic lung diseases | 302 (10.0) | 158 (12.6) | 68 (17.9) | *<*0.001 |
| Liver disease | 136 (4.5) | 40 (3.2) | 16 (4.2) | 0.141 |
| Heart disease | 356 (11.8) | 195 (15.5) | 59 (15.5) | 0.002 |
| Stroke | 49 (1.6) | 50 (4.0) | 10 (2.6) | *<*0.001 |
| Kidney disease | 176 (5.8) | 75 (6.0) | 22 (5.8) | 0.982 |
| Digestive disease | 690 (22.9) | 296 (23.6) | 98 (25.8) | 0.430 |
| Psychiatric disease | 33 (1.1) | 14 (1.1) | 4 (1.1) | 0.994 |
| Memory-related disease | 34 (1.1) | 22 (1.8) | 8 (2.1) | 0.123 |
| Arthritis or rheumatism | 1026 (34.0) | 508 (40.5) | 144 (37.9) | *<*0.001 |
| Asthma | 111 (3.7) | 69 (5.5) | 24 (6.3) | 0.005 |
| Anaemia^a^ | 444 (17.2) | 213 (20.6) | 94 (31.4) | *<*0.001 |
| Handgrip strength, kg | 31.7 ± 8.3 | 22.1 ± 8.4 | 22.2 ± 6.9 | *<*0.001 |
| ASM/Ht^2^, kg/m^2^ | 7.2 ± 1.5 | 7.5 ± 1.0 | 5.6 ± 2.3 | *<*0.001 |
| Total cognitive score | 14 (10, 18) | 12 (7, 16) | 11 (7, 15) | *<*0.001 |

Data are shown as means ± standard deviation, median (interquartile range), or numbers (percentages).

^a^ Missing data: 218 for smoking, 672 for drinking, 448 for educational level, 1777 for socioeconomic status, and 740 for anaemia data.

Abbreviation: ASM, appendicular skeletal muscle; BMI, body mass index.

**Supplementary Table 3. Cross-sectional association between low muscle mass alone and depressive symptoms** **in CHARLS 2015.**

|  | **Cases, n (%)** | **OR (95% CI)** | |
| --- | --- | --- | --- |
|  |  | **Model 1^a^** | **Model 2^b^** |
| Muscle mass (n=4310) |  |  |  |
| Reference (n=3543) | 738 (20.8) | 1 | 1 |
| Low muscle mass alone (n=767) | 189 (24.6) | 0.99 (0.75, 1.30) | 0.94 (0.69, 1.29) |

Abbreviation: OR, Odds ratio. Reference was a group of the participants without any sarcopenia components. Low muscle mass alone: low muscle mass with neither low grip strength nor slow physical performance. **^a^** Model 1 was adjusted for age, sex, residence, marital status, educational level, smoking status, drinking status, socioeconomic status and body mass index. **^b^** Model 2 was adjusted as model 1 with further adjustment for comorbidities, anaemia and total cognitive score.
